# MELAS syndrome presenting as an acute surgical abdomen

**DOI:** 10.1308/003588414X13824511649733

**Published:** 2014-01

**Authors:** S Dindyal, K Mistry, N Angamuthu, G Smith, D Hilton, Arumugam P, J Mathew

**Affiliations:** ^1^Whittington Hospital NHS Trust,UK; ^2^Royal Cornwall Hospitals NHS Trust,UK

**Keywords:** MELAS, Mitochondrial disease, Encephalomyopathy, Acute abdomen, Lactic acidosis, Stroke-like episodes

## Abstract

MELAS (mitochondrial cytopathy, encephalomyopathy, lactic acidosis and stroke-like episodes) is a syndrome in which signs and symptoms of gastrointestinal disease are uncommon if not rare. We describe the case of a young woman who presented as an acute surgical emergency, diagnosed as toxic megacolon necessitating an emergency total colectomy. MELAS syndrome was suspected postoperatively owing to persistent lactic acidosis and neurological symptoms. The diagnosis was later confirmed with histological and genetic studies. This case highlights the difficulties in diagnosing MELAS because of its unpredictable presentation and clinical course. We therefore recommend a high index of suspicion in cases of an acute surgical abdomen with additional neurological features or raised lactate.

MELAS syndrome (mitochondrial cytopathy, encephalomyopathy, lactic acidosis and stroke like episodes) is an uncommon, progressive, neurodegenerative disease in which gastrointestinal involvement is a rare feature. We describe the case of a young woman who presented to our accident and emergency department with an acute surgical abdomen. Based on clinical and imaging findings, the differential diagnoses for this patient included severe colitis and toxic megacolon. An emergency total colectomy was performed and histology confirmed ischaemic colitis. Postoperatively, persistent lactic acidosis and encephalopathy prompted further neurological evaluation. Subsequent intestinal and muscle biopsy as well as genetic studies confirmed the diagnosis of MELAS syndrome. This case report highlights the uncommon presentation, diagnostic difficulties and significant morbidity associated with this disease.

## Case history

A 34-year-old Caucasian woman presented to our casualty department with sudden onset of lower abdominal pain and vomiting of 12 hours’ duration. Clinical examination revealed diffuse tenderness with rebound signs in the lower abdomen. She went on to have an episode of collapse from which she was resuscitated and taken to the intensive care unit (ICU), sedated, intubated and ventilated. She had complained of several episodes of abdominal pain over the last few weeks and was treated by her general practitioner for irritable bowel. Her past medical history included depression, for which she took fluoxetine 20mg daily. Four years previously, magnetic resonance imaging of the brain was performed for persistent galactorrhoea, which ruled out a pituitary mass but showed basal ganglia calcification; she was also diagnosed with bilateral neurosensory loss.

Initial haematology and biochemical results were: haemoglobin 19.6g/dl, white cell count 42.1 × 10^9^/l, amylase 110iu/l, C-reactive protein 15mg/l and corrected calcium 3.67. Computed tomography (CT) of the abdomen revealed a markedly dilated colon and rectum with thickening of its wall and enhancement suggestive of toxic colitis. Normal enhancement of the major mesenteric arteries was noted.

In the ICU, the patient was severely acidotic with blood gas analysis showing a pH 7.09, lactate levels of 15mmol/l and a base excess of 22. This started to normalise with large amounts of fluid. She maintained a good urine output, and her tachycardia and blood pressure normalised. Diarrhoea developed 12 hours after admission. Subsequent flexible sigmoidoscopy showed ischaemic mucosa from the rectosigmoid junction to the descending colon. At this time, a diagnosis of acute colitis or toxic megacolon secondary to ulcerative colitis was made.

**Figure 1:**
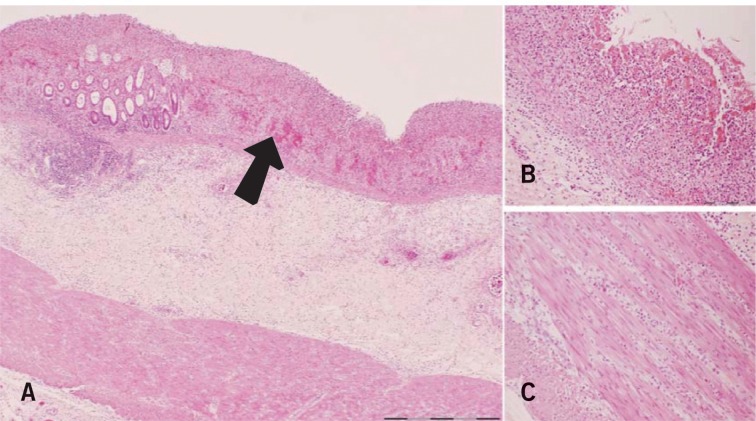
Histological appearances of the colon: partial to full-thickness (arrow) ischaemic mucosal necrosis and submucosal oedema (×10 magnification) (A); haemorrhagic necrosis of the mucosa in areas (×20 magnification) (B), and extension of inflammation and oedema into the muscularis propria (×20 magnification) (C)

The following day the patient underwent a laparotomy and was found to have an ischaemic colon; an emergency total colectomy and end ileostomy was performed. The rectal stump was stapled and anchored in the subcutaneous area. Histological examination showed extensive ischaemic change in the colonic wall with minimal inflammation and normal blood vessels (Fig 1). The postoperative period was characterised by persistent lactic acidosis and a decreased level of consciousness (despite withdrawal of sedation) requiring ventilator support for 12 days.

**Figure 2:**
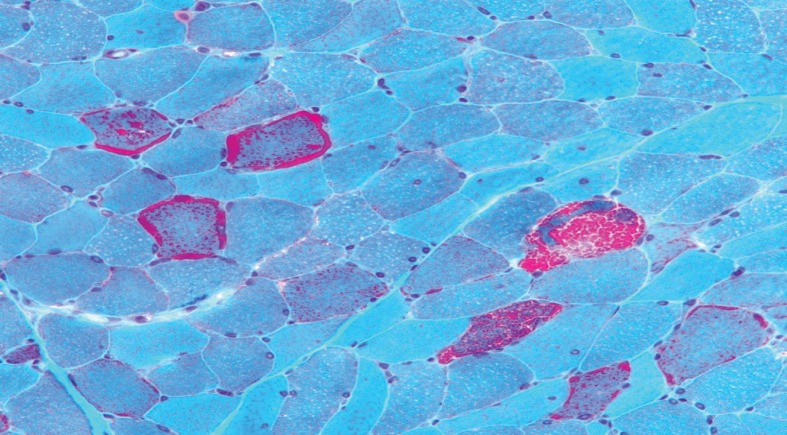
Gömöri trichrome preparation showing scattered ‘ragged red’ fibres

The triad of ischaemic colitis, lactic acidosis and abnormal brain imaging prompted a neurology review. The possibility of a mitochondrial disorder was considered based on the postoperative events, previous deafness and calcification of basal ganglia. CT of the brain revealed extensive calcification of basal ganglia, periventricular calcification and calcification extending into the cerebellum; diffuse involutional changes were noted in the ventricles and sulci. A subsequent skeletal muscle biopsy revealed ragged red fibres (Fig 2), cytochrome c oxidase-negative fibres, succinate dehydrogenase hyperreactive blood vessels and paracrystalline arrays in mitochondria. Genetic studies revealed an A3243G mutation characteristic of MELAS syndrome.

The patient stayed in the ICU for 55 days and was sent to a rehabilitation ward for intensive physiotherapy. She presented seven months later with sudden onset of central abdominal pain and vomiting, and was managed conservatively for adhesive partial bowel obstruction.

## Discussion

Mitochondrial cytopathies are a diverse group of inherited and acquired disorders of the mitochondria. About 80% of patients with the clinical features of MELAS syndrome exhibit a heteroplasmic A3243 mutation.^1^ The diagnostic criteria for MELAS include:^2^

- stroke-like episodes
- encephalopathy with seizures and dementia
- mitochondrial myopathy characterised by lactic acidosis, ragged red fibres on muscle biopsy, accumulation of abnormal mitochondria in smooth muscles and endothelial cells as well as mutations of mitochondrial deoxyribonucleic acid detected at genetic testing

Sensorineural hearing loss, non-insulin dependent diabetes mellitus, urinary retention and recurrent headaches are other recognised features of MELAS syndrome that have been documented. There have been a few reports in the literature of MELAS associated with gastrointestinal involvement such as recurrent vomiting, anorexia, colitis, severe constipation, paralytic ileus and colonic volvulus.^3–5^ However, it is very rare for cases of MELAS syndrome to present initially with gastrointestinal symptoms. Surgical intervention in MELAS has been required occasionally in patients with ischaemic colitis,^3^ segmental ileal paralysis, pseudo-obstruction, gastric perforation and megacolon.

## Conclusions

Clinicians managing patients with either acute or chronic gastrointestinal symptoms not attributable to a specific cause should maintain a high index of suspicion for mitochondrial cytopathy. Recurrent vomiting, diarrhoea, pseudo-obstruction or an acute abdomen associated with lactic acidosis or neurological symptoms should prompt an urgent review of the patient with MELAS syndrome in mind. Histological and genetic evaluation needs to be carried out to confirm the diagnosis. The prognosis is unfavourable, especially in patients with sepsis and metabolic disturbances undergoing surgery, as encephalomyopathy and cardiac involvement in MELAS is associated with a high morbidity and mortality.
